# Supplementary material for: Economic evaluation of antimicrobial resistance in curable sexually transmitted infections; a systematic review and a case study
Source: PLoS One. 2023 Oct 19;18(10):e0292273. doi: 10.1371/journal.pone.0292273 (PMC10586702; doi:10.1371/journal.pone.0292273)
Supplement: S1 File — (DOCX) [file pone.0292273.s001.docx]

# S1 Table

## Search strategy

MEDLINE

|  | **Searches** |
| --- | --- |
| 1 | exp Sexually Transmitted Diseases, Bacterial/ |
| 2 | ((bacteria* or parasitic or curable or cure*) adj2 ('sexually transmitted disease*' or 'sexually transmitted infection* or STI or STD)).mp. |
| 3 | exp Neisseria gonorrhoeae/ |
| 4 | exp Gonorrhea/ |
| 5 | (gonorrhoea* or gonorrhea*).mp. |
| 6 | exp Chlamydia trachomatis/ |
| 7 | exp CHLAMYDIA/ |
| 8 | chlamydia*.mp. |
| 9 | exp Treponema pallidum/ |
| 10 | exp SYPHILIS/ |
| 11 | (syphilis or treponema).mp. |
| 12 | exp Trichomonas Infections/ |
| 13 | (trichomoniasis or trichomonas).mp. |
| 14 | exp Mycoplasma genitalium/ |
| 15 | Mycoplasma genitalium.mp. |
| 16 | or/1-15 |
| 17 | exp Drug Resistance, Multiple, Bacterial/ |
| 18 | exp Drug Resistance, Microbial/ |
| 19 | exp Drug Resistance, Bacterial/ |
| 20 | exp Treatment Failure/ |
| 21 | exp Anti-Bacterial Agents/ec [Economics] |
| 22 | (treatment* adj2 fail*).mp. |
| 23 | (super or resist* or untreatable or susceptib* or sensitiv*).mp. |
| 24 | (AMR or ABR or MDRGC).ti,ab. |
| 25 | or/17-24 |
| 26 | exp ECONOMICS/ |
| 27 | exp Cost-Benefit Analysis/ |
| 28 | exp "Costs and Cost Analysis"/ |
| 29 | exp Health Care Costs/ |
| 30 | exp Economics, Dental/ |
| 31 | exp Economics, Hospital/ |
| 32 | exp Economics, Medical/ |
| 33 | exp Economics, Nursing/ |
| 34 | exp Economics, Pharmaceutical/ |
| 35 | exp "Cost of Illness"/ |
| 36 | (economic$ or cost or costs or costly or costing or price or prices or pricing or pharmacoeconomic$).mp. |
| 37 | (decision adj (analy$ or model$ or tree$)).mp. |
| 38 | exp Quality-Adjusted Life Years/ |
| 39 | (quality-adjusted life year$ or QALY$).ti,ab,kw. |
| 40 | (expenditure$ not energy).ti,ab. |
| 41 | value for money.ti,ab. |
| 42 | budget$.mp. |
| 43 | or/26-42 |
| 44 | letter.pt. |
| 45 | editorial.pt. |
| 46 | historical article.pt. |
| 47 | or/44-46 |
| 48 | 43 not 47 |
| 49 | Animals/ |
| 50 | Humans/ |
| 51 | 49 not (49 and 50) |
| 52 | 48 not 51 |
| 53 | 16 and 25 and 52 |

Embase

|  | **Searches** |
| --- | --- |
| 1 | exp bacterial infection/ or (bacteria* or curable).mp. |
| 2 | exp sexually transmitted disease/ |
| 3 | 1 and 2 |
| 4 | ((bacteria* or parasitic or curable or cure*) adj2 ('sexually transmitted disease*' or 'sexually transmitted infection* or STI or STD)).mp. |
| 5 | exp Neisseria gonorrhoeae/ |
| 6 | exp gonorrhea/ |
| 7 | (gonorrhoea* or gonorrhea*).mp. |
| 8 | exp Chlamydia trachomatis/ |
| 9 | exp Chlamydia/ |
| 10 | chlamydia*.mp. |
| 11 | exp Treponema pallidum/ |
| 12 | exp syphilis/ |
| 13 | (syphilis or treponema).mp. |
| 14 | exp Trichomonas/ |
| 15 | (trichomoniasis or trichomonas).mp. |
| 16 | exp Mycoplasma genitalium/ |
| 17 | Mycoplasma genitalium.mp. |
| 18 | or/3-17 |
| 19 | exp antibiotic resistance/ |
| 20 | exp multidrug resistance/ |
| 21 | exp penicillin resistance/ |
| 22 | exp treatment failure/ |
| 23 | exp antiinfective agent/pe [Pharmacoeconomics] |
| 24 | (treatment* adj2 fail*).mp. |
| 25 | (super or resist* or untreatable or susceptib* or (sensitiv* adj2 (drug or antimicrobial or antibiotic or antibacterial))).mp. |
| 26 | (AMR or ABR or MDRGC).ti,ab. |
| 27 | or/19-26 |
| 28 | exp economics/ |
| 29 | exp "cost benefit analysis"/ |
| 30 | exp "cost effectiveness analysis"/ |
| 31 | exp "cost utility analysis"/ |
| 32 | exp "cost minimization analysis"/ |
| 33 | exp "cost of illness"/ |
| 34 | exp "health care cost"/ |
| 35 | exp "hospital cost"/ |
| 36 | exp economic aspect/ |
| 37 | exp health economics/ |
| 38 | (economic$ or cost or costs or costly or costing or price or prices or pricing or pharmacoeconomic$).mp. |
| 39 | (decision adj (analy$ or model$ or tree$)).mp. |
| 40 | exp quality adjusted life year/ |
| 41 | (quality-adjusted life year$ or QALY$).ti,ab,kw. |
| 42 | (expenditure$ not energy).ti,ab. |
| 43 | value for money.ti,ab. |
| 44 | budget$.mp. |
| 45 | or/28-44 |
| 46 | letter.pt. |
| 47 | editorial.pt. |
| 48 | historical article.pt. |
| 49 | or/46-48 |
| 50 | 45 not 49 |
| 51 | animal/ |
| 52 | human/ |
| 53 | 51 not (51 and 52) |
| 54 | 50 not 53 |
| 55 | 18 and 27 and 54 |

Cochrane Library

|  | **Searches** |
| --- | --- |
| 1 | ((bacteria* or parasitic or curable or cure*) adj2 ("sexually transmitted disease*" or "sexually transmitted infection*" or STI or STD)).mp. |
| 2 | (gonorrhoea* or gonorrhea*).mp. |
| 3 | chlamydia.mp. |
| 4 | syphilis.mp. |
| 5 | treponema pallidum.mp. |
| 6 | (trichomoniasis or trichomonas).mp. |
| 7 | mycoplasma genitalium.mp. |
| 8 | #1 or #2 or #3 or #4 or #5 or# 6 or #7 |
| 9 | (antibiotic resistance) |
| 10 | (multidrug resistance) |
| 11 | (penicillin resistance) |
| 12 | (treatment failure) |
| 13 | (antiinfective agent) |
| 14 | ((treatment* adj2 fail*)) |
| 15 | ((AMR or ABR or MDRGC)):ti,ab,kw |
| 16 | #9 OR #10 OR #11 OR #12 OR #13 OR #14 OR #15 |
| 17 | (economics) |
| 18 | ("cost benefit analysis") |
| 19 | ("cost effectiveness analysis") |
| 20 | ("cost utility analysis") |
| 21 | ("cost minimization analysis") |
| 22 | ("cost of illness") |
| 23 | ("health care cost") |
| 24 | ("hospital cost") |
| 25 | (economic aspect) |
| 26 | (health economics) |
| 27 | ((economic$ or cost or costs or costly or costing or price or prices or pricing or pharmacoeconomic$)) |
| 28 | ((decision adj (analy$ or model$ or tree$))) |
| 29 | (quality adjusted life year) |
| 30 | ((quality-adjusted life year$ or QALY$)):ti,ab,kw |
| 31 | ((expenditure$ not energy)):ti,ab,kw |
| 32 | (value for money):ti,ab,kw |
| 33 | (budget$) |
| 34 | #17 OR #18 OR #19 OR #20 OR #21 OR #22 OR #23 OR #24 OR #25 OR #26 OR #27 OR #28 OR #29 OR #30 OR #31 OR #32 OR #33 |
| 35 | #8 AND #16 AND #34 |

Health Technology Assessment database, National Health Service Economic Evaluation Database and EconLit

|  | **Searches** |
| --- | --- |
| 1 | ((bacteria* or parasitic or curable or cure*) adj2 ("sexually transmitted disease*" or "sexually transmitted infection*" or STI or STD)).mp. |
| 2 | (gonorrhoea* or gonorrhea*).mp. |
| 3 | chlamydia.mp. |
| 4 | syphilis.mp. |
| 5 | treponema pallidum.mp. |
| 6 | (trichomoniasis or trichomonas).mp. |
| 7 | mycoplasma genitalium.mp. |
| 8 | or/1-7 |
