# Supplementary material for: Economic evaluation of antimicrobial resistance in curable sexually transmitted infections; a systematic review and a case study
Source: PLoS One. 2023 Oct 19;18(10):e0292273. doi: 10.1371/journal.pone.0292273 (PMC10586702; doi:10.1371/journal.pone.0292273)
Supplement: S2 File — (DOCX) [file pone.0292273.s002.docx]

## S2 File

# Study selection

In stage I, studies were categorised into the following groups (A-G) on the basis of title and abstract:

1. The study involves a formal economic evaluation of treatments for curable STIs based on primary and/or secondary data (e.g. previously published studies or other sources) and considers AMR in the analysis;
2. The study involves a formal economic evaluation of treatments for curable STIs based on primary and/or secondary data, however, it is unclear if AMR is considered in the analysis;
3. The study discusses economic aspects of AMR in relation to the treatment of curable STIs and contains relevant primary and/or secondary data;
4. The study discusses costs associated with curable STIs, however, it is unclear if AMR is considered in the analysis;
5. Unclear if the study falls under (A), (B), (C), or (D) but may be a potentially useful review;
6. The study is not relevant to the economic evaluation of treatments for curable STIs;
7. Copy/duplicate publication.

Full text of studies categorised as A-C were carried forward to stage II. Studies in categories D-G were excluded. Full texts were further classified into the following groups:

1. Economic evaluation of treatment for curable STIs with consideration of AMR (incorporation of AMR in the analysis or explicit acknowledgement/justification for the disregard of AMR);
2. Cost study with assessment of the cost of AMR in relation to curable STIs;
3. Other study with some assessment of cost implications of AMR in relation to curable STIs or reporting relevant outcomes which may serve as a proxy for costs;
4. Review/editorial of economic aspects of AMR in relation to curable STIs;
5. Economic evaluation or cost study in curable STIs with no consideration of the cost implications of AMR;
6. Foreign language publication;
7. Not relevant to the economic evaluation of treatments for curable STIs;
8. Full text unavailable.

Studies classified in groups 1-3 were included for quality assessment and form the final evidence base for the review.
