# Supplementary material for: Economic evaluation of antimicrobial resistance in curable sexually transmitted infections; a systematic review and a case study
Source: PLoS One. 2023 Oct 19;18(10):e0292273. doi: 10.1371/journal.pone.0292273 (PMC10586702; doi:10.1371/journal.pone.0292273)
Supplement: S3 File — (DOCX) [file pone.0292273.s003.docx]

**S4 File-Quality assessment of included studies**

**Table1 Quality assessment of cost studies using the cost of illness checklist**

| **Item no.** | **Question** | **Answers** | | | | |
| --- | --- | --- | --- | --- | --- | --- |
|  |  | **CDC 2015** | **Turner 2017** | **Allan-Blitz 2018** | **Chesson 2018** | **Wynn and Klausner 2020** |
| 1 | Was the illness of interest defined? | Y | Y | Y | Y | Y |
| 2 | Was the analysis limited to a defined population? | Y | Y | Y | Y | Y |
| 3 | Was the setting described in which the costs occur? | N | Y | Y | Y | Y |
| 4 | Was the study design described? | N | Y | Y | Y | Y |
| 5 | Was the study perspective stated? | N | Y | Y | N | Y |
| 6 | Was the time horizon of costs stated? | Y | N | N | Y | NR |
| 7 | Was the method for resource use identification described? | N | Y | Y | Y | Y |
| 8 | Were relevant resources omitted with regards to the chosen study perspective? | NR | N | Y | NR | Y |
| 9 | Was a justification given for omitting relevant resources with regard to the chosen perspective? | NR | N | Y | NR | Y |
| 10 | If indirect costs are included in analysis, were productivity losses clearly reported separately? | NR | NR | NR | NR | NR |
| 11 | Were the data sources used for the measurement of resources reported? | N | Y | Y | Y | Y |
| 12 | Were data sources adjusted for the German context? | NA | NA | NA | NA | NA |
| 13 | Was an analysis performed to account for missing data? | NR | NR | NR | NR | NR |
| 14 | Were the resources valued according to the chosen study perspective? | NR | Y | Y | P | Y |
| 15 | Were all data sources used for the valuation of resources reported? | N | Y | Y | Y | Y |
| 16 | Was the method for valuation of productivity losses reported? | NR | NR | NR | NR | NR |
| 17 | Were costs discounted and discount rates reported? | NR | N | N | Y | N |
| 18 | Were cost data adjusted for inflation and, if so, are the methods specified? | NR | NR | Y | NR | NR |
| 19 | Were resources valued according to a specific year (index year)? | NR | Y | Y | Y | Y |
| 20 | Were price adjustments considered over a period of time? | NR | N | N | Y | N |
| 21 | Were unit prices adopted from other countries? | NR | N | N | N | N |
| 22 | Were data evaluation methods described comprehensively/transparently? | N | Y | Y | Y | Y |
| 23 | Were subgroup analyses performed? | N | Y | N | N | N |
| 24 | Were sensitivity analysis performed? | N | N | N | Y | Y |
| 25 | Were sensitivity analyses performed for resource use or unit prices? | N | N | N | Y | Y |
| 26 | Were parameters listed or discussed that could be subject to uncertainty? | N | N | Y | Y | Y |
| 27 | Was modelling used to estimate costs? | NR | Y | Y | Y | Y |
| 28 | Was the quantity of resources used listed separately from valued costs? | N | Y | N | Y | Y |
| 29 | Is the derivation of cost calculations comprehensible/transparent? | N | N | P | Y | Y |
| 30 | Were different cost categories listed separately? | N | Y | Y | Y | Y |
| 31 | Are the conclusions comprehensible? | Y | Y | Y | Y | Y |
| 32 | Was the quality of the data source(s) discussed? | N | Y | Y | Y | Y |
| 33 | Were study results compared to findings from other studies? | N | Y | Y | Y | Y |
| 34 | Were study limitations discussed or reported? | N | Y | Y | Y | Y |
| 35 | Did the study discuss generalisability of the study results? | N | Y | Y | Y | Y |
| 36 | Were recommendations given for future research? | N | Y | Y | Y | NR |
| 37 | Does the article indicate any potential conflict of interest? | NR | Y | NR | NR | NR |
| Total |  | Y=4, N=19, NR=13, P=1, NA=1 | Y=22, N=10, NR=4, NA=1 | Y=23, N=8, NR=4, P=1, NA=1 | Y=25,N=3,NR=7, NA=1 | Y=25, N=4, NR=7, NA=1 |

Abbreviations: N, no; NA, not applicable; NR, not reported; P, partially; Y, yes

**Table 2 Quality assessment of economic evaluations using an adapted CHEERS checklist**

| **Item no.** | **Section/item** | **Recommendation** | **Reported on page number** | | | | | | | | | | | | | |
| --- | --- | --- | --- | --- | --- | --- | --- | --- | --- | --- | --- | --- | --- | --- | --- | --- |
|  |  |  | **Phillips 1989** | | **Nettleman 1990** | | **Crabbe 2000** | | **Roy 2005** | | **Price 2006** | | **Xiridou 2016** | | **Harding-Esch 2020** | |
| **Title & abstract** | | | | | | | | | | | | | | | | |
| 1 | Title | Identify the study as an economic evaluation, or use more specific terms such as ‘‘cost-effectiveness analysis’’ and describe the interventions compared. | p.297, type of analysis not specified | | p.175 | | p.640 title specifies CEA | | p.1265, type of analysis not specified | | p.202, title specifies CEA | | p.542, title specifies CEA not CUA | | p.1, title specifies CEA | |
| 2 | Abstract | Provide a structured summary of objectives, perspective, setting, methods (including study design and inputs), results (including base-case and uncertainty analyses), and conclusions. | p.297 | | p.175, brief, no structure | | p.640 | | p.1265, no structure | | p.202 | | p.542 | | p.1 | |
| **Introduction** | | | | | | | | | | | | | | | |  |
| 3 | Background & objectives | Provide an explicit statement of the broader context for the study. Present the study question and its relevance for health policy or practice decisions. | p.297 | p.175, very brief | | p.640-641 | | p.1265 | | p.202 | | p.542 | | p.1-2 | |  |
| **Methods** | | | | | | | | | | | | | | | |  |
| 4 | Target population & sub-groups | Describe characteristics of the base-case population and subgroups analysed including why they were chosen | p.297 | p.176 | | p.641, no sub-groups | | p.1265 | | p.202-203 | | p.542 | | p.3 | |  |
| 5 | Setting & location | State relevant aspects of the system(s) in which the decision(s) need(s) to be made. | p.297 | p.175-176 | | NR | | p.1265 | | p.202 | | p.542 | | p.3 | |  |
| 6 | Study perspective | Describe the perspective of the study and relate this to the costs being evaluated. | NR | NR | | NR | | p.1265, abstract | | p.204 | | p.544 | | p.4 | |  |
| 7 | Comparators | Describe the interventions or strategies being compared and state why they were chosen. | p.297-298 | p.177 | | p.641 | | p.1265-1266, Table 1 | | p.203 | | p.543-544 | | p.2-4 | |  |
| 8 | Time horizon | State the time horizon(s) over which costs and consequences are being evaluated and say why appropriate. | p.299 | NR | | NR | | NR | | NR | | p.544 | | p.4 | |  |
| 9 | Discount rate | Report the choice of discount rate(s) used for costs and outcomes and say why appropriate. | p.299 | NR | | NR | | NR | | NR | | p.544 | | NR | |  |
| 10 | Choice of health outcomes | Describe what outcomes were used as the measure(s) of benefit in the evaluation and their relevance for the type of analysis performed. | NR | p.177, reports only one outcome | | p.643 | | p.1266 | | p.205 | | p.544 | | p.3 | |  |
| 11a | Measurement of effectiveness | Single study–based estimates: Describe fully the design features of the single effectiveness study and why the single study was a sufficient source of clinical effectiveness data. | NR | NR | | NR | | - | | - | | - | | - | |  |
| 11b |  | Synthesis-based estimates: Describe fully the methods used for the identification of included studies and synthesis of clinical effectiveness data. | NR | NR | | NR | | p.1266-1267, Table 2 | | p.203, Table 1 | | p.544, Table 1 | | p.3, Table2 | |  |
| 12 | Measurement and valuation of preference-based outcomes | If applicable, describe the population and methods used to elicit preferences for outcomes. | NA | NR | | NA | | NA | | NA | | p.544, limited details | | NA | |  |
| 13a | Estimating costs & resources | Single study–based economic evaluation: Describe approaches used to estimate resource use associated with the alternative interventions. Describe primary or secondary research methods for valuing each resource item in terms of its unit cost. Describe any adjustments made to approximate to opportunity costs. | - | - | | - | | - | | - | | - | | - | |  |
| 13b |  | Model-based economic evaluation: Describe approaches and data sources used to estimate resource use associated with model health states. Describe primary or secondary research methods for valuing each resource item in terms of its unit cost. Describe any adjustments made to approximate to opportunity costs. | p.298-299, Table 1 | p.176-177 | | p.641-643 | | p.1267-1268, Table 3 | | p.204-205, Table 1 | | p.544, Table 2 | | p.4, Table3 | |  |
| 14 | Currency, price date, and conversion | Report the dates of the estimated resource quantities and unit costs. Describe methods for adjusting estimated unit costs to the year of reported costs if necessary. Describe methods for converting costs into a common currency base and the exchange rate. | p.300 | NR | | NR | | p.1268 | | p.204 | | p.544 | | p.4 | |  |
| 15 | Choice of model | Describe and give reasons for the specific type of decision-analytic model used. Providing a figure to show model structure is strongly recommended. | p.298, Fig 1 | NR, model type not specified | | p.641-644, Fig. 1/2/3, no rationale | | p.1266, diagram in Fig. A1 appendix | | p.205, no diagram | | p.543, Fig. S1/S2 suppl. material | | p.3, suppl. fig S1 | |  |
| 16 | Assumptions | Describe all structural or other assumptions underpinning the decision-analytic model. | p.298-299 | p.176-177 | | p.641 | | p.1265-1268 | | p.203-205 | | p.543-544 | | p.2-3 | |  |
| 17 | Analytic methods | Describe all analytic methods supporting the evaluation. This could include methods for dealing with skewed, missing, or censored data; extrapolation methods; methods for pooling data; approaches to validate or make adjustments (e.g., half-cycle corrections) to a model; and methods for handling population heterogeneity and uncertainty | p.299 | NR | | NR | | NR | | p.205 | | p.543-544 | | p.3-4 | |  |
| **Results** | | | | | | | | | | | | | | | |  |
| 18 | Study parameters | Report the values, ranges, references, and if used, probability distributions for all parameters. Report reasons or sources for distributions used to represent uncertainty where appropriate. Providing a table to show the input values is strongly recommended. | p.298-299, text only | p.177, Table 1, no table for costs | | p.644, Table 1 | | p.1267-1268, Table 2/3 | | p.203, Table 1 | | p.543, Table 1 | | p.2-3,Table 3 | |  |
| 19 | Incremental costs & outcomes | For each intervention, report mean values for the main categories of estimated costs and outcomes of interest, as well as mean differences between the comparator groups. If applicable, report incremental cost-effectiveness ratios. | NR | NR | | p.644, Table 2, ICERs not reported | | p.1269, Table 4, ICERs in Table A1 appendix | | p.205, Table 3 | | p.545, Table 3 | | NR | |  |
| 20a | Characterising uncertainty | Single study–based economic evaluation: Describe the effects of sampling uncertainty for estimated incremental cost, incremental effectiveness, and incremental cost-effectiveness, together with the impact of methodological assumptions (such as discount rate, study perspective). | - | - | | - | | - | | - | | - | | - | |  |
| 20b |  | Model-based economic evaluation: Describe the effects on the results of uncertainty for all input parameters, and uncertainty related to the structure of the model and assumptions. | p.300 | NR | | p.644-645, Table 3 | | p.1270-1271, Table 4 | | p.206, Table 4 | | p.545-547,  Fig. 2/3 | | p.5, suppl. fig 2-5 | |  |
| 21 | Characterising heterogeneity | If applicable, report differences in costs, outcomes, or cost-effectiveness that can be explained by variations between subgroups of patients with different baseline characteristics or other observed variability in effects that are not reducible by more information. | NR | NR | | NR | | p.1269-1271, Table 4/5, Fig. 1/2 | | NR | | p.545-546, Fig. 1 | | Table 5 | |  |
| **AMR-specific methods and results** | | | | | | | | | | | | | | | |  |
| 22 | AMR-specific methods and results | Clearly state the type of resistance considered in the analysis and provide a precise definition. | p.299 | NR | | NR | | NR | | NR | | p.543 | | p.2, Table 1 | |  |
| 23 |  | Clearly state any assumptions regarding AMR and provide justification for their inclusion. | p.299 | p.176, not justified | | p.644, Table 1, not clearly stated | | p.1267 | | p.203 | | p.545 | | p.3, suppl. Table S1 | |  |
| 24 |  | Report the impact of varying key assumptions regarding AMR as tested in sensitivity analyses. | p.300 | p.178 | | p.644-645 | | p.1269-1271 | | p.206-207, Table 4 | | p.545-546 | | p.6-7, suppl. fig s2-6 | |  |
| **Discussion** | | | | | | | | | | | | | | | |  |
| 25 | Study findings, limitations, generalisability, and current knowledge | Summarize key study findings and describe how they support the conclusions reached. Discuss limitations and the generalizability of the findings and how the findings fit with current knowledge. | p.300-301 | p.178-179 | | p.645-646 | | p.1271-1273 | | p.206-208 | | p.547-548 | | p.7-10 | |  |
| 26 | Source of funding | Describe how the study was funded and the role of the funder in the identification, design, conduct, and reporting of the analysis. Describe other nonmonetary sources of support. | NR | NR | | NR | | NR | | p.202, sources of support | | NR | | p.10, funding details provided | |  |
| 27 | Conflicts of interest | Describe any potential for conflict of interest among study contributors in accordance with journal policy. In the absence of a journal policy, we recommend authors comply with International Committee of Medical Journal Editors’ recommendations. | NR | p.175 | | NR | | NR | | NR | | p.542 | | p.10 | |  |

Abbreviations: AMR, antimicrobial resistance; CEA, cost-effectiveness analysis; ICER, incremental cost-effectiveness ratio; NA, not applicable; NR, not reported.
