# Supplementary material for: Economic evaluation of antimicrobial resistance in curable sexually transmitted infections; a systematic review and a case study
Source: PLoS One. 2023 Oct 19;18(10):e0292273. doi: 10.1371/journal.pone.0292273 (PMC10586702; doi:10.1371/journal.pone.0292273)
Supplement: S4 File — (DOCX) [file pone.0292273.s004.docx]

# S5 File

## Assumptions for the economic evaluation comparing gentamicin to ceftriaxone for the treatment of gonorrhoea

The following assumptions were made;

1. The cost of other equipment used in treatment (syringes etc.) were excluded because, the use of these equipment would have be similar in both arms.
2. In both trial arms patients received a single oral dose of azithromycin, an initial consultation with a health care professional and a follow-up visit. These costs were excluded from the analysis as they were incurred equally across the trial arms.
3. We also assumed that the delivery of the treatment and length of consultation were similar across treatment arms for the base case analysis. This was informed by a survey on treatment nurses. Majority of whom (17/21 respondents, 81%) indicated that there was no difference in the time taken to administer the two treatments. However, increased time for gentamicin treatment was explored as part of the sensitivity analysis.
4. At the 2-week gonorrhoea test of cure, if infection was not cleared at all sites (as indicated by a NAAT test), further treatment was given. For the initial analysis, where the infection was not cleared, patients in the gentamicin arm were assumed to be treated with ceftriaxone (unless there was a contraindication for this treatment). Patients in the ceftriaxone arm were assumed to have been given a second course of ceftriaxone unless the microbial culture demonstrated resistance to this treatment. This was to reflect the guidelines at the time [1] (Table 4).
5. In the secondary analyses accounting for AMR, alternative scenarios were explored for persistent infections at the 2-week test of cure. These included the use of gentamicin treatments if the infection was resistant to ceftriaxone.

## References

1. Bignell C, Fitzgerald M. UK national guideline for the management of gonorrhoea in adults, 2011. Int J STD AIDS. 2011;22(10):541-7. Epub 2011/10/15. doi: 10.1258/ijsa.2011.011267. PubMed PMID: 21998172.
