# Supplementary material for: Economic evaluation of antimicrobial resistance in curable sexually transmitted infections; a systematic review and a case study
Source: PLoS One. 2023 Oct 19;18(10):e0292273. doi: 10.1371/journal.pone.0292273 (PMC10586702; doi:10.1371/journal.pone.0292273)
Supplement: S1 Table — (DOCX) [file pone.0292273.s005.docx]

# S3 Table

## Data extraction form

|  |  |
| --- | --- |
| Identification features of article |  |
|  | Author |
|  | Article Title |
|  | Citation |
|  | Type of publication |
|  | Country of Origin |
| Article characteristics | |
|  | Study aim |
|  | Study objectives |
|  | Study design |
|  | Study inclusion criteria |
|  | Study exclusion criteria |
|  | Sample size |
|  | Study duration |
|  | Type and source of cost |
| Participant characteristics | |
|  | Mean/median age of participants |
|  | Gender |
|  | Sexual orientation |
|  | Type of STI |
|  | Infection site |
| Intervention | |
|  | Intervention and setting |
|  | Number of participants in intervention group |
|  | STI intervention |
|  | length of follow-up |
| Comparator | |
|  | Comparator and setting |
|  | Number of participants in comparator group |
|  | STI comparator |
|  | length of follow-up |
|  | Comparator |
| Outcome |  |
|  | Method used to assess the cost burden of AMR (provide definition or reference to method, if available) |
|  | How was AMR defined |
|  | What assumptions were made for AMR |
|  | How was AMR incorporated |
|  | What was the rationale for AMR exclusion |
|  | Reported cost/economic outcomes |
|  | Base case health outcomes (e.g. QALYs) |
|  | Base case ICER |
|  | Base case total costs |
|  | Summary of results from sensitivity analyses including impact of incorporation of AMR on results if reported |
|  | Direct (medical and non-medical) costs associated with AMR in the context of curable STIs—Cost study |
|  | Indirect costs associated with AMR in the context of curable STIs—cost study |
|  | Study conclusions with focus on impact of AMR on cost-effectiveness results if reported |
|  | Proxy Outcomes |
| Model |  |
|  | Model Structure |
|  | Model Perspective |
|  | Model Time horizon |
|  | Model—Half cycle correction |
|  | Perspective of analysis of costs—cost study |
|  | Discounting |
| Study Limitations |  |
