# Supplementary material for: Economic evaluation of antimicrobial resistance in curable sexually transmitted infections; a systematic review and a case study
Source: PLoS One. 2023 Oct 19;18(10):e0292273. doi: 10.1371/journal.pone.0292273 (PMC10586702; doi:10.1371/journal.pone.0292273)
Supplement: Attachment — Submitted filename: NG AMR Review - Response to reviewers 2023 07 17.docx [file pone.0292273.s006.docx]

We thank the reviewers for their comments and suggestions. We have revised the manuscript to reflect these and have provided specific responses below.

**Reviewer #1:** Congratulations for selecting a research topic of great interest with results that may have potential implications for clinical practice and public health policies.
The systematic review methodology follows current evidence based practice, is well described and comprehensively documented in tables and supplementary files.
For PLOS ONE readers is highly relevant to be aware that there are no consistent methodological approaches in various economic analyses which could lead to variable results and thus recommendations. It is instructive to learn about the parameters influencing the results which are well described and summarised in this paper.
Maybe the conclusions part could a bit more powerful with stronger messages on the next steps in this research area.

**Response:** We thank the reviewer for their comment. We made conclusions based on the identified need for more developmental research to inform approaches to capture and incorporate AMR into economic evaluations. We apologise if this was not initially clear and have strengthened the conclusions as follows (lines 499-510) ‘*There is dearth of data in relation to the economic analysis of AMR for curable STIs. However, by incorporating AMR costs (including in our exemplar study on alternative treatment for gonorrhoea) more robust interpretations in relation to the costs/cost effectiveness of new or alternative treatment and strategies can be made. The current evidence relating to the economic impact of AMR for curable STIs is generally limited to direct, aggregated costs over a short period, obtained from high income countries, is largely specific to gonorrhoea AMR, and often lacks a societal perspective. There is no standardised approach for the measurement of AMR in patients with curable STIs which results in uncertainty when reporting on the economic impact of AMR and makes comparisons between studies difficult. Further research is required to inform guidance on optimal approaches to capture AMR costs for curable STIs (e.g. how AMR is defined and how AMR associated costs are adequately captured) and methodically incorporate such costs into economic evaluations.*’

**Reviewer #2:** Overall this is a very comprehensive study with significant clinical and public health indications. The authors conducted a systematic review of the economic evaluation for STI treatment (gonorrhoea) by incorporating antimicrobial resistance (AMR), which is topical.

However, they have only identified a small number of studies. The majority of the studies are conducted either in the USA or UK. After 63 pages the only conclusion is “although ceftriaxone was the cheaper treatment, gentamicin became cost-neutral if the clinical efficacy of ceftriaxone reduced from 98% to 92%. I am unsure how these findings can be interpreted for low-middle-income countries particularly after accounting for the exchange rates.

Nevertheless given the limited information on this topic, I believe the current study will bring insight into the economic implications of AMR on treating STIs.

**Response:** We thank the reviewer for their comment and agree that the current evidence base is limited as we have highlighted in lines 433-4434 (‘*we found a small number of studies were eligible for inclusion, suggesting that the existing evidence base is limited’*). This is why, in addition to the systematic review, we explored various approaches to incorporate AMR costs using prospectively collected data from a large RCT. Although most published evidence originated from high income countries as did our case-study, we discuss the likely implications for LMIC settings in relation to the cost of AMR (lines 437-440, ‘*Cost estimates from the United States indicate that the cost of gonorrhoea resistance to current first-line treatment (ceftriaxone) is potentially substantial, equating to $235 to $378.2 million in direct medical costs over 10 years [36, 38]. These estimates are likely to be significantly higher in low and middle income settings [18]*’). We also highlight the limitations of the review (lines 502-505, *‘The current evidence relating to the economic impact of AMR for curable STIs is generally limited to direct, aggregated costs over a short period, obtained from high income countries, is largely specific to gonorrhoea AMR, and often lacks a societal perspective*’). We have added further comment in respect to our case study economic evaluation in lines 495-498, ‘*Also, as our case study was conducted in a high income setting, the findings may not be generalizable to low income settings but contributes to the debate on how future economic evaluations could more fully incorporate the economic impact of AMR for curable STIs.*
